# Can broad-spectrum multinutrients treat symptoms of antenatal depression and anxiety and improve infant development? Study protocol of a double blind, randomized, controlled trial (the ‘NUTRIMUM’ trial)

**DOI:** 10.1186/s12884-020-03143-z

**Published:** 2020-08-25

**Authors:** Hayley A. Bradley, Siobhan A. Campbell, Roger T. Mulder, Jaqueline M. T. Henderson, Lesley Dixon, Joseph M. Boden, Julia J. Rucklidge

**Affiliations:** 1 School of Psychology, Speech and Hearing, University of Canterbury, Private Bag 4800, Christchurch, 8041 New Zealand; 2 Department of Psychological Medicine, University of Otago, Christchurch, New Zealand; 3 New Zealand College of Midwives, Christchurch, New Zealand

**Keywords:** Antenatal, Perinatal, Pregnancy, Depression, Anxiety, Treatment, Intervention, Micronutrients, Infant development

## Abstract

**Background:**

Untreated antenatal depression and anxiety can be associated with short and long term health impacts on the pregnant woman, her infant and the rest of the family. Alternative interventions to those currently available are needed. This clinical trial aims to investigate the efficacy and safety of a broad-spectrum multinutrient formula as a treatment for symptoms of depression and anxiety in pregnant women and to determine the impact supplementation has on the general health and development of the infant.

**Methods:**

This randomised, controlled trial will be conducted in Canterbury, New Zealand between April 2017 and June 2022. One hundred and twenty women aged over 16 years, between 12 and 24 weeks gestation and who score ≥ 13 on the Edinburgh Postnatal Depression Scale (EPDS) will be randomly assigned to take the intervention (*n* = 60) or an active control formula containing iodine and riboflavin (*n* = 60) for 12 weeks. After 12 weeks, participants can enter an open-label phase until the birth of their infant and naturalistically followed for the first 12 months postpartum. Infants will be followed until 12 months of age. Randomisation will be computer-generated, with allocation concealment by opaque sequentially numbered envelopes. Participants and the research team including data analysts will be blinded to group assignment. The EPDS and the Clinical Global Impressions Scale of Improvement (CGI-I) will be the maternal primary outcome measures of this study and will assess the incidence of depression and anxiety and the improvement of symptomatology respectively. Generalized linear mixed effects regression models will analyse statistical differences between the multinutrient and active control group on an intent-to-treat basis. A minimum of a three-point difference in EPDS scores between the groups will identify clinical significance. Pregnancy outcomes, adverse events and side effects will also be monitored and reported.

**Discussion:**

Should the multinutrient formula be shown to be beneficial for both the mother and the infant, then an alternative treatment option that may also improve the biopsychosocial development of their infants can be provided for pregnant women experiencing symptoms of depression and anxiety.

**Trial registration:**

Trial ID: ACTRN12617000354381; prospectively registered at Australian New Zealand Clinical Trials Registry on 08/03/2017.

## Background

Depression and anxiety are the most common mental health problems during pregnancy and are amongst the leading causes of maternal morbidity and mortality worldwide [1]. Internationally, rates of antenatal depression and anxiety range between 3.5–18.4% [2, 3] and 6.6–24.7% [3, 4] respectively. In New Zealand, rates of antenatal depression and anxiety appear to be higher than this with between 12 and 22% of pregnant women experiencing depression [5, 6] and 20–25% suffering from anxiety [5]. Antenatal depression and anxiety are highly comorbid and begin in or continue into pregnancy. The signs and symptoms are typically the same as those for depression and anxiety in the non-pregnant population [7].

Antenatal depression and anxiety is associated with short and long-term health consequences for not only the pregnant women affected but the infant and rest of the family. Pregnancy and neonatal complications such as hypertension, preeclampsia, and gestational diabetes [8]; low birth weight, foetal growth restriction [9, 10] and preterm birth [11–13] are more likely in individuals who experience antenatal depression and anxiety. Depression and anxiety during pregnancy have also been associated with poor cognitive, emotional and behavioural development of the infant [12, 14–16], poor maternal attachment to the infant and increased risk of depression in partners [15, 16].

The Royal Australian and New Zealand College of Psychiatrists recommend the use of pharmacological interventions such as antidepressants as the first line of treatment to address severe depression and anxiety during pregnancy, despite the lack of evidence supporting the efficacy of its use [17] and the reluctance of women to use this form of treatment during pregnancy [18–20].

Over the last few years, retrospective observational studies have been increasingly recognizing the risks associated with in-utero exposure to antidepressants including congenital malformations [21, 22], poor neonatal adaptation syndrome [23, 24] and persistent pulmonary hypertension in the infant [25, 26]. Offspring exposed to antidepressant medication during pregnancy are also at greater risk of neurodevelopmental delay [27–31], impairments to language skills [32] and of developing depression during adolescence [33]. Although recent studies have concluded that foetal exposure to antidepressant medication poses no risk to developing an autism spectrum disorder (ASD) or attention-deficit/hyperactivity disorder (ADHD) [33–35], most found that the incidence of these disorders were two fold prior to controlling for confounding variables. For all pregnant women suffering from depression and/or anxiety, the risks of exposing the foetus to antidepressant medication during pregnancy must be weighed against the risks to both the mother and the foetus of untreated depression and/or anxiety during the antenatal period [36].

The first line of treatment recommended for mild to moderate depression and anxiety during pregnancy is psychological interventions such as cognitive-behavioural therapy and interpersonal therapy (IPT) [17]. Although evidence from open trials and RCTs to support the efficacy of IPT for depression [37, 38] and open trials of CBT and mindfulness for anxiety [39]are increasing and the majority of pregnant women prefer this form of treatment over medication, many have difficulty accessing these treatments due to issues with time, stigma, cost and childcare [40].

It is evident that current treatments for depression and anxiety during the antenatal period may not be either accessible or acceptable to many women struggling with these symptoms during pregnancy. Alternative interventions that are safe, acceptable and more accessible therefore need to be identified.

Nutritional interventions present a possible treatment option for psychological symptoms presenting during pregnancy. Indeed, evidence is emerging from cohort and cross-sectional studies suggesting that diet plays a significant role in maternal mental health during the antenatal period where poor diet quality has been associated with symptoms of antenatal depression, anxiety and stress [41] and a healthy dietary pattern has been associated with lower symptoms [42] and an inverse association with antenatal and postnatal anxiety and prenatal depression [43]. Furthermore, a recent systematic review of cross sectional and cohort studies found that a traditional Japanese and UK diet during pregnancy was associated with a lower risk of prenatal depression and anxiety respectively [43]. The review found no significant association between a Western dietary pattern and symptoms of perinatal depression and anxiety.

It is well known that a poor diet can lead to nutritional deficiencies and indeed, pregnancy and lactation is a period where nutritional deficiencies are more likely to arise given the increased demands on the pregnant woman’s body. The pregnant woman therefore is at a greater risk of developing nutritional deficiencies. Some studies investigating the association between blood concentration of various nutrients and mental health have linked lower serum levels of nutrients such as vitamin D, zinc, folate and vitamin B12 with increased depressive symptoms [44–47] as well as selenium, fats and fatty acids, whereas other have found no association [48, 49]. There is currently insufficient evidence on deficiencies to predict who is at risk of developing antenatal depression and anxiety and as such, more research is needed.

While it is important to investigate the impact of nutrients individually, it is rare for nutritional deficiencies to occur in isolation and instead, given that they are not consumed singularly in the diet, deficiencies in multiple micronutrients are more likely [50]. Nutrients are required for the synthesis of neurotransmitters and interact synergistically and antagonistically with each other for proper functioning of the mind and body, as such, a combined approach is likely more appropriate [51]. Indeed, evidence has accumulated over the last 10 years showing that broad spectrum multinutrient interventions may provide larger effects for a variety of psychological conditions including depression, anxiety, and ADHD relative to any single nutrient alone [52].

While a number of studies have focused on the prevention of postnatal depression using micronutrient supplementation, investigations for efficacious treatments of antenatal depression and anxiety has been relatively neglected until recently. Only two studies have been conducted to date that assist with determining whether nutrients can alter the course of depression during pregnancy in women who are symptomatic. A randomised controlled trial examined the effects of omega 3 fatty acids compared with a placebo in women diagnosed with a major depressive disorder during pregnancyand found that the omega 3 fatty acids had a greater effect on reducing depression scores relative to placebo [53]. A second randomized controlled trial conducted in Vietnam found that women scoring in the highest tertile for depressive symptoms (as measured by the CES-D) at preconception, had lower depression scores during the first and second trimesters of pregnancy after taking multiple micronutrients or a combined iron-folic acid formula during the preconception period than women who consumed a folic acid only formula [54].

To date, no studies have been conducted examining the efficacy of nutritional supplements for the treatment of antenatal anxiety in women who are symptomatic. More specifically, no study has yet addressed whether broad-spectrum multinutrients (vitamins and minerals taken in combination) is an efficacious treatment for women who experience symptoms of depression and anxiety during pregnancy. This study therefore aims to investigate the efficacy and safety of nutrients in a pregnant population experiencing such symptoms. This study also aims to offer some etiological and mechanistic insight into the relationship between multinutrients and mood by examining levels of certain nutrients and biomarkers of inflammation such as homocysteine and cytokines as predictors and mediators of treatment response. Indeed, concentrations of various pro-inflammatory cytokines have been found to be higher in the depressed adult population [55] and emerging evidence is finding a link between antenatal depression and elevated levels of inflammation; however, the findings are mixed [56]. Nevertheless, studies in support of the inflammatory theory have found a relationship between antenatal depression and an increased risk of various foetal and maternal morbidities such as preeclampsia, preterm birth, gestational diabetes which are known to be associated with increased inflammation [56].

Given that nutrients serve an important role in the structural and functional development of the foetus [57], this study will also examine the impact multinutrient exposure in utero has on infant development. Indeed, deficiencies in certain nutrients and/or an unhealthy diet during pregnancy have been associated with congenital malformations, poor pregnancy, labour and birth outcomes and impaired cognitive [58], long-term emotional behavioural development [59] and hyperactivity-inattention symptoms [59]. While there is limited evidence for the association between maternal diet and beneficial outcomes for offspring, the observational study, KOMCHS in Japan showed that behavioural problems in childhood were less likely in children whose mothers consumed a diet high in vegetables, fruit and vitamin C during pregnancy [60] and used supplements of folate, vitamin B2 and vitamin B6 [61]. A Danish cohort study (DNBC) reported the risk for hyperkinetic disorders and treatment for ADHD to be lower in children whose mothers used multivitamins early in pregnancy compared to those who consumed folic acid only [62]. There have been limited studies to date examining the impact of diet and dietary supplementation on infants whose mothers experienced mental health difficulties during the antenatal period.

Supplementation of certain nutrients such as folic acid, vitamin A and vitamin D at specific time points during the antenatal period has been associated with improved physical health and emotional development outcomes in childhood and reduced likelihood of later mental illnesses [63, 64]. For supplementation of a broad-spectrum multinutrients, evidence from the developing world has shown a reduced risk of low birth weight and found no significant adverse effects overall in infants exposed to multinutrients in utero [65, 66]. A birth cohort study conducted in China found that prenatal multinutrient supplementation compared to iodine-folic acid supplementation was associated with increased scores of communication, gross motor, fine motor, problem-solving and personal-social skills in offspring at 36 months old [67]. In a follow up study of an RCT in Nepal where access to food is limited, infants exposed to a multi-vitamin and mineral supplement in utero were found to have a higher birth weight and increased body weight/size at 2 years of age than infants exposed to an iron and folic acid supplement [68]. The effects of multinutrient supplementation on these outcomes is unknown in infants whose mothers experienced mental health difficulties during the antenatal period.

It seems plausible that supplementation with a broad range of nutrients during pregnancy may also be of benefit to the foetus and infant by improving foetal growth and biopsychosocial outcomes. This study therefore additionally aims to explore and begin to document the health and developmental outcomes of infants exposed to multinutrients in-utero.

### Aims

This trial aims to investigate the efficacy and safety of a broad-spectrum multinutrient formula compared to an active control formula for the treatment of symptoms of antenatal depression and anxiety using a double blind randomized controlled design. We anticipate that women randomized to receive the multinutrient formula will show a greater improvement in depressive and anxiety symptoms compared to women taking the active control formula. We hypothesise that improvements will generalise to secondary measures of sleep, stress, emotion regulation and quality of life.

An exploratory aspect of this trial aims to document potential effects of an enriched nutrient environment during utero on infant outcomes. As such, we will explore whether length of exposure to nutrients is a predictor of infant temperamental outcomes. We hypothesise that a more nutrient-rich foetal environment will contribute to more positive long-term health and developmental outcomes for the infant.

## Methods/design

### Trial design

This randomized, double-blind, controlled trial is designed to compare the efficacy and safety of a broad-spectrum multinutrient formula to an active control formula for the treatment of depressive and anxiety symptoms in pregnant women, allocated in a 1:1 ratio. The 12 week RCT phase of the trial will be followed by an open label phase where participants can take the multinutrient intervention until the birth of their infant. The open label phase was included in order to provide participants with the opportunity to try the nutrients regardless of the random allocation in the RCT phase without breaking the blind. Participants will be followed up naturalistically until 12 months postpartum.

### Study setting

The trial will be conducted at the University of Canterbury, Christchurch, New Zealand. Enrolment of the first participant occurred on 12 April 2017; the last participant is anticipated to enrol by December 2020.

### Eligibility criteria

Pregnant women who are between 12 and 24 weeks gestation will be recruited and followed throughout their pregnancy until 12 months postpartum. The inclusion criteria are aged ≥16 years; low risk singleton pregnancy; free from psychiatric medication for 4 weeks; score of ≥13 on the Edinburgh Postnatal Depression Scale (EPDS) during their second trimester and deemed reliable and compliant with the protocol. The exclusion criteria include regular vomiting; current/recent significant pregnancy complications (e.g. placenta praevia, preeclampsia, gestational diabetes), known foetal abnormalities; serious current or historical medical condition (e.g. hypertension, kidney disease); known allergy to the ingredients of the intervention; known metabolic condition (e.g. Wilson’s disease, hemochromatosis); untreated or unstable thyroid disease; known neurological disorder (e.g. epilepsy, multiple sclerosis, narcolepsy); desire to continue taking prenatal supplements that either exceed the upper limit or are not required for medical purposes (decisions discussed and made on a case-by-case basis).

All participants will be invited to enrol their infant in the study examining infant development over the first 12 months of life.

### Intervention

Participants will be randomly assigned to receive either a multinutrient formula (Daily Essential Nutrients (DEN)) or an active control formula containing iodine and riboflavin for a 12-week period in order to allow enough time for the treatment to reach a substantial effect. Iodine is recommended to be taken throughout the duration of pregnancy in New Zealand and will therefore be used as an active control so that an additional iodine supplement will not need to be taken alongside the study interventions.

The full dose of 12 capsules will be taken as four capsules three times daily. Titration to the full dose will occur over a 7-day period whereby participants take one capsule, three times each day with the dose increasing by three capsules every second day until the maximum dose is achieved. Taken at the full dose, both formulas provide the daily supplemental intake of iodine for pregnant and lactating women as recommended by the New Zealand Ministry of Health. The dose of iodine in the active control formula will be distributed across 12 capsules such that participants randomized to the active control condition consume the same number of capsules. The multinutrient formula contains riboflavin (vitamin B_2_), a vitamin known to colour the urine. Riboflavin will be added to the active control formula to mimic the possible effect on urine colour in order to maintain the double-blind.

Both the multinutrient and the active control formulas are similar in appearance and will be taken orally in capsule form. They will be packaged in white opaque bottles containing two vanilla sachets to ensure all the capsules smell the same. Each bottle will have a label identifying the participant number. Both formulas are suitable for participants with intolerances or allergies to gluten, dairy and soy. The full list of ingredients and doses can be viewed in Table 1.

**Table 1:** Intervention ingredients

**Daily Essential Nutrients Supplement Facts**	
Amount per serving (12 capsules)	
Vitamin A (as retinyl palmitate)	5760 IU
Vitamin C (as ascorbic acid)	600 mg
Vitamin D (as cholecalciferol)	3000 IU
Vitamin E (as d-alpha tocopheryl succinate)	360 IU
Vitamin K (75% as phylloquinone; 25% as menaquinone-7)	120 mcg
Thiamin (as thiamin mononitrate)	60 mg
Riboflavin	18 mg
Niacin (as niacinamide)	90 mg
Vitamin B6 (as pyridoxine hydrochloride)	69.9 mg
Folate (as L-methylfolate calcium)	801 mcg
Vitamin B12 (as methylcobalamin)	900 mcg
Biotin	1080 mcg
Pantothenic acid (as d-calcium pantothenate)	30 mg
Calcium (as chelate)	1320 mg
Iron (as chelate)	13.8 mg
Phosphorus (as chelate)	840 mg
Iodine (as chelate)	204 mcg
Magnesium (as chelate)	600 mg
Zinc (as chelate)	48 mg
Selenium (as chelate)	204 mcg
Copper (as chelate)	7.2 mg
Manganese (as chelate)	9.6 mg
Chromium (as chelate)	624 mcg
Molybdenum (as chelate)	144 mcg
Potassium (as chelate)	240 mg
Proprietary blend: Choline bitartrate, Alpha-lipoic acid, Mineral wax, Inositol, Acetyl-L-carnitine, Grape seed extract, *Ginkgo biloba* leaf extract, L-methionine, N-acetyl-L-cysteine, Boron (as chelate), Vanadium (as chelate), Lithium orotate (as chelate), Nickel (as chelate) Other ingredients: Vegetarian capsule (hypromellose), Microcrystalline cellulose, Magnesium stearate, Silicon dioxide, Titanium dioxide	
**Active Control Supplement Facts**	
Amount per serving (12 capsules)	
Riboflavin	1.2 mg
Potassium Iodide	150 mcg
Silicon dioxide	30 mg
Magnesium stearate	60 mg
Microcrystalline cellulose	6 g

DEN was chosen for this study as the combination of nutrients it contains is the most studied combination in the world for the treatment of psychiatric illnesses [51] and has found robust effects on a range of psychological symptoms that are also implicated in antenatal depression and anxiety [52, 69–72]. Furthermore, the safety and tolerability of this combination of multinutrients is well documented in both adults and children [72, 73].

During the RCT phase of the trial, participants will be able to continue taking omega 3 and probiotic supplements if they were taking these prior to screening for the trial. Participants will also be able take iron, if needed, at any time during the pregnancy and will be able to continue any form of psychotherapy that had begun prior to screening. Participants will be withdrawn from the study if they are prescribed and take any psychiatric medication given the potential interaction with nutrients. Participants will be encouraged before enrolment in the trial to avoid taking additional nutrients, other than those needed for medical reasons, throughout the duration of the trial in order to minimize the risk of adverse events. However, they may be withdrawn from the trial if the dose of a prescribed vitamin or mineral supplement exceeds the upper limit. Decisions on continued participation in the trial will be made on a case-by-case basis. The upper limit is the maximum dose of a nutrient that can be taken without any adverse effects.

Following the 12-week RCT period, all participants will be invited to enter an open label phase until the birth of their infants and where the capsule dose will be identical and follow the same titration procedure as the RCT phase. The product is commercially available for participants to purchase should they wish to continue taking the multinutrients post-trial.

### Outcomes

**Fig. 1:**
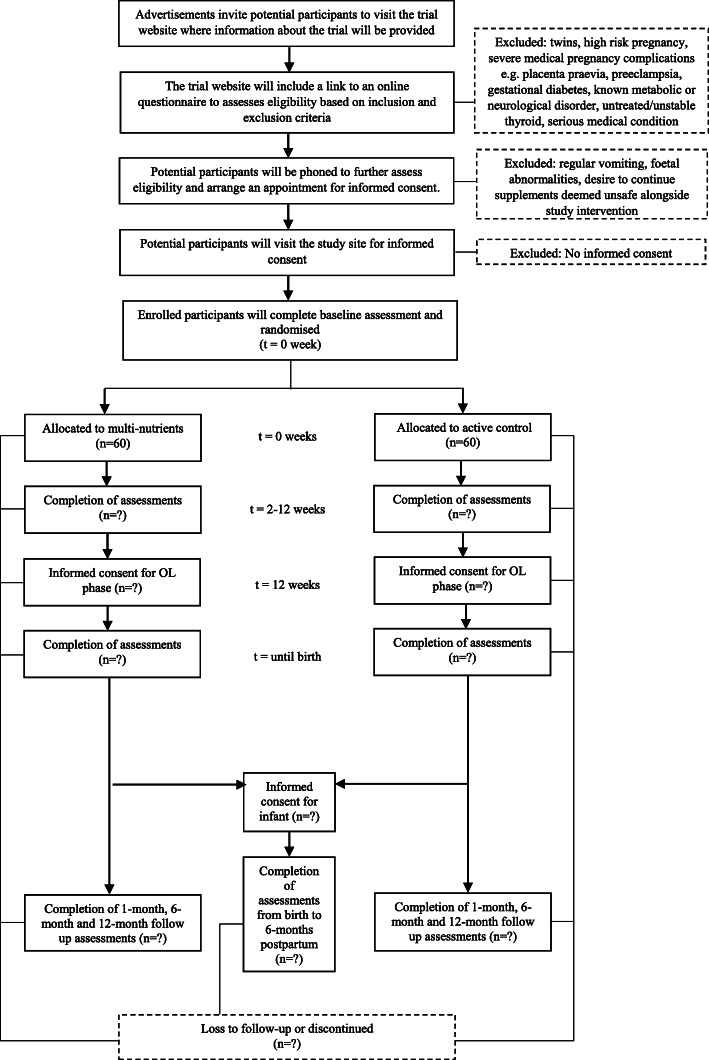
SPIRIT Flow Diagram: Schedule of Enrolment, Intervention, and Assessments

Figure 1 displays the outcome measures of the study and the time points at which each measure will be administered. Demographic characteristics including age, ethnicity, education, occupation, household income, number in household, children and marital status will be collected in a screening assessment. All self-report measures will be independently completed by participants via the web-based data collection system, Qualtrics.

#### Maternal primary outcome measures

The *Edinburgh Postnatal Depression Scale (EPDS)* [74] is a 10 item self-report questionnaire designed to assess the cognitive and affective symptoms of depression and anxiety over the previous 7 days. The EPDS is the most widely used screening questionnaire for postpartum depression [75] in both research and clinical practice and has also been validated as a screening measure for probable depression during pregnancy [76–78]. Each item on the EPDS is rated on a four-point scale, with a total score ranging from 0 to 30, with higher scores indicating greater levels of distress. A cut off score of 13 has been found to have the optimum specificity and sensitivity in detecting an incidence of major depression in the postnatal period [74]; however, cut off scores during the antenatal period are controversial and culturally dependent; ranging from 4.5 to 13.5 [79]. Despite disagreements in the cut off score for the incidence of major depression during the antenatal period, a cut off score of ≥13 will be used in this study to identify the incidence of perinatal depression. A cut-off score of ≥13 has been used in prevalence studies in New Zealand [5, 6] and has been shown to not miss any women in need of help for their mood in Australian clinical practice [80]. A cut off score of 10 has been shown to be optimal to identify the presence of minor depression in postnatal populations [74]. For this purpose and given the variability of cut-off scores to identify antenatal depression in particular, the present study will use a score of 10–12 to identify minor depression and anxiety and ≤ 9 to identify the absence of perinatal depression and anxiety.

The *Clinical Global Impressions – Improvement Scale (CGI-I)* [81] is a clinician-rated assessment of the change in participant’s symptoms from baseline ranging from 1 (very much improved) to 7 (very much worse). The CGI-I will be administered by graduate students under the supervision of a clinical psychologist and will assess mood, anxiety and global improvements. Participants enrolled in the clinical trial will be asked to rate their own improvement in mood, anxiety and globally at the end of the 12-week RCT phase and again every 4 weeks during the OL phase. The CGI-I has demonstrated high external validity (r = 0.74) [82] and is widely used in clinical trials.

#### Maternal secondary outcome measures

The *Clinical Global Impressions – Severity Scale (CGI-S)* [81] is a clinician-rated evaluation of symptom severity ranging from 1 (normal, not ill) to 7 (very severely ill). The CGI-S is commonly used in clinical trials and in a routine clinical setting, has shown to be sensitive to clinical change in diagnostically diverse populations including those diagnosed with depression and anxiety [83]. The CGI-S will be administered by graduate students under the supervision of a clinical psychologist and will assess the severity of mood, anxiety and global functioning.

*The Montgomery and Asberg Depression Rating Scale (MADRS)* [84] is a 10 item clinician-rated scale that assesses the frequency, severity and duration of depressive symptoms over the previous week. Each item is rated on a 7-point scale ranging from 0 to 5, with a maximum score of 60. A greater score indicates more severe symptoms. The MADRS demonstrates excellent reliability and good validity [84].The MADRS will be administered by psychology graduate students using the Structured Interview Guide for the Montgomery-Asberg Depression Rating Scale (SIGMA) [85] under the supervision of a clinical psychologist. The SIGMA has demonstrated good to excellent interrater reliability and intraclass correlation (r = 0.93) [85].

*Depression Anxiety Stress Scale 21 (DASS21)* [86] is a 21 item self-report questionnaire with three, seven item subscales measuring the severity of depression, anxiety and stress, each summing to a maximum score of 21 with elevated scores indicating more severe symptoms. Each item on the DASS-21 is rated on a four point scale ranging from 0 (did not apply to me at all) to 3 (applied to me very much, or most of the time). The DASS-21 provides clinical cut-off scores for each subscale ranging from normal to extremely severe. The DASS-21 has been widely used in both clinical and non-clinical populations and has demonstrated strong validity and internal consistency (α = 0.95 depression, 0.90 anxiety, 0.93 stress, 0.97 total score) [87].

*Generalized Anxiety Disorder – 7 item (GAD-7)* [88] *is* a 7-item self-report measure of generalised anxiety symptoms over the past 2 weeks. Each item is rated on a 4-point scale ranging from 0 (not at all) to 3 (nearly every day). Total scores range from 0 to 21 with higher scores indicating more severe symptoms. The GAD-7 includes the following cut off scores: normal: 0–4; mild: 5–9; moderate: 10–14 and severe: 15–21. The GAD-7 has demonstrated good internal consistency and test-retest-reliability for diagnosing generalised anxiety disorder in the general population [88, 89]. In perinatal populations, the GAD-7 has yielded a sensitivity of 61.3% and specificity of 72.7% at a cut off score of 13 [90].

*Perceived Stress Scale (PSS)* [91] is a 10 item self-report questionnaire measuring an individual’s experience of stress on a 5-point scale ranging from 0 (never) to 4 (very often). Total scores range from 0 to 40 with higher scores indicating greater levels of stress. The PSS includes cut off scores to indicate the severity of stress: low: 0–13; moderate: 14–26; high: 27–40. The PSS has established test-retest reliability and internal consistency (α= > 0.70) in culturally diverse populations including pregnancy [92].

*Short Form Health Survey – 12 version 1 (SF-12v1)* [93] a 12 item self-report questionnaire assessing physical and mental components of health related quality of life over the previous 4 week period. Total scores from each component ranges from 0 to 100 with higher scores indicating better quality of life. The SF-12 has demonstrated good reliability and validity in the general population [93] and for a variety of severe mental health diagnoses [94]. The SF-12 has demonstrated excellent internal consistency in both the first (α = 0.83) [95] and third (α = 0.84) trimester of pregnancy [96].

*Difficulties in Emotion Regulation Scale – Short Form (DERS-SF)* [97] is an 18 item self-report questionnaire assessing emotion dysregulation across six subscales which identify lack of awareness, clarity, acceptance, access to strategies, engagement in goal-directed activities and ability to manage impulses when experiencing negative emotions. Cronbach’s alpha coefficient for each subscale ranged from 0.75 to 0.92 in an adult population [98].

*Global Assessment of Functioning (GAF)* [99] is a clinician-rated scale included in the DSM-III-R, that considers the severity of social, psychological and occupational functioning of mental health not due to physical or environmental limitations. The GAF is measured on a scale of 0–100, a higher score indicating a higher level of functioning and includes the following cut-off scores: normal: 91–100; mild: 61–90; moderate: 31–60; severe: 0–30. The GAF has demonstrated excellent reliability (ICC = 0.92) [100].

*The Antidepressant Side-Effect Checklist (ASEC)* [101] is a list of 21 symptoms that assesses side effects of the intervention. The ASEC asks participants to indicate the severity of their symptoms on a four-point scale ranging from 0 (absent) to 3 (severe). The ASEC is a self-report assessment of side effects and has been adapted to include further symptoms including rash, itching and numbness, pain, burning or tingling in hands, feet or legs associated with vitamin toxicity. The ASEC is well used and validated in antidepressant drug trials and has been adapted for use with multinutrients. Interrater reliability of each item on the ASEC ranges from 0.55 to 0.89 and internal consistency was adequate ranging from 0.77 to 0.78 [101].

*The Pittsburgh Sleep Quality Index (PSQI)* [102]*)* is a 19 item self-report questionnaire that assesses sleep quality, sleep latency, sleep duration and disturbance, habitual sleep efficiency, sleep medication usage and daytime dysfunction over the past month. Each item is rated on a four point scale ranging from 0 to 3 with “poor” sleep indicated at a sum score of five or more [102]. In pregnancy cohorts, the PSQI has demonstrated good construct validity [103] and internal consistency (α = 0.69–0.81) [103–105].

*Treatment Satisfaction Questionnaire for Medication – 11 (TSQM-11)* [106] *is* an 11 item self-report questionnaire assessing how satisfied participants are with using the multinutrients and the effect of the multinutrients on three domains: effectiveness, convenience and global satisfaction. Each item is rated on a seven point scale ranging from extremely dissatisfied to extremely satisfied with total scores ranging from 0 to 100; higher scores indicating greater satisfaction. The TSQM-11 has demonstrated internal consistency (α = 0.84–0.94) and high test-retest reliability (ICC= > 0.7) [106].

*Biomarkers* of inflammation (interleukin- 4, interleukin- 6, interleukin- 10 and tumor necrosis factor-α) and nutrients (vitamin B12, vitamin D, zinc, copper, ferritin, iron, homocysteine, ceruloplasmin, folate) will be analysed from blood samples. A haematology blood count will also be performed.

#### Maternal covariate outcome measures

Additional outcomes will be used to assess any mediating or moderating impacts on the treatment outcome.

The *Multidimensional Scale of Perceived Social Support (MSPSS)* [107] is a 12 item self-report scale measuring the subjective adequacy of social support from family, friends and significant others over the previous 8 week period. Items on the MSPSS are rated on a seven-point scale ranging from one to seven with higher scores indicative of greater social support. The MSPSS has demonstrated good internal and test-retest reliability and moderate construct validity [107]. In the pregnant population, the MSPSS has shown excellent internal consistency (α = 0.90–0.92) [104, 108].

*Stressful Life Events Questionnaire (SLEQ)* [109] is a 26 item self-report questionnaire assessing the incidence of stressful life events. The scale will be modified for the purposes of this study to exclude the item “you suffered from a mental illness” and include the following items: “You moved house”, “You got married”, “You had a pet go missing, die or have you had to rehome it”. The SLEC was developed for and originally used in a study examining the impact of maternal stress during pregnancy on cognitive development and fearfulness in infancy [109].

*Heaviness of Smoking Index (HIS)* [110] is a two item self-report questionnaire assessing a person’s level of nicotine dependence with higher scores indicating a more severe dependence. For the purposes of using an online assessment tool, the question “Over the past two weeks, have you smoked a cigarette?” will be added to identify whether or not participants have used cigarettes. The HIS has shown to good reliability (r = 0.72–0.70) and predictive validity, whereby higher scores have been associated with subsequent quit attempts [111].

*The AUDIT - Consumption (AUDIT-C)* [112] is a 3 item self-report questionnaire to assess alcohol consumption and identify hazardous drinkers or active alcohol use problems. Items are rated on five-point scale (0–4) with total scores ranging from 0 to 12; higher scores indicating alcohol consumption as a greater risk to safety. A score of three or more has demonstrated the optimal sensitivity (0.66) and specificity (0.94) for identifying alcohol dependency or abuse [112]. In pregnancy populations, the AUDIT-C has shown to be the most sensitive tool for identifying alcohol dependency or abuse (0.71) with excellent sensitivity (0.98) and high specificity (0.85) [113].

*Drug Abuse Screening Test - 10 (DAST-10)* [114] *is* a 10 item self-report questionnaire assessing drug use and dependence and has been adapted for the purposes of this study. Participants who identify to have used drugs over each two-week period will be asked questions 1, 3 and 9 of the DAST-10, which have demonstrated a Cronbach’s alpha of 0.55, 0.69 and 0.78 respectively. Participants will also be asked to identify what substances they have used.

*Dietary Screening Tool (DST)* [115] is a 24 item self-report questionnaire that assesses dietary intake and identifies those at nutritional risk. The DST has a total score of 100 with higher scores indicating a healthier dietary pattern. The DST has yielded good sensitivity (0.83) and specificity (0.75). The DST has been adapted to include New Zealand foods for the purposes of this study.

*Dietary Inflammatory Index (DII)* [116] lists 45 foods and nutrients that positively or negatively affect levels of inflammation. Data from a three-day food diary developed for the purposes of this study will be used to calculate a DII with higher scores indicating greater anti-inflammatory effects of foods.

*The Helping Alliance Questionnaire (HAQ)* is a five item self-report questionnaire developed for the purposes of the study to assess the extent to which the participant has an alliance with the assessor. Each item is rated on a 6 point scale from 0 (strongly disagree) to 5 (strongly agree) with higher scores indicating a greater alliance.

*The Dutch Measure for quantification of Treatment Resistance in Depression (DM-TRD)* [117] is a refined and extended staging measure of the Maudsley Staging Method [118] used to predict treatment resistance, severity of future depressive symptoms and remission. The DM-TRD will be scored based on outcomes from the SCID-5RV, GAF, Standardised Assessment of Personality – Abbreviated Scale (SAPAS) [119] and questions developed for this study to assess past treatment and therapy.

*Anthropometry.* Participants height, weight and blood pressure (systolic, diastolic and pulse) will be taken throughout the trial.

#### Infant measures

The *Brazelton Neonatal Behavioural Assessment Scale (NBAS)* [120] is a comprehensive assessment of both neurological integrity and behavioural functioning. The NBAS will be administered by a graduate student to assess the full range of infant neurobehavioral performance (orientation to auditory and visual stimuli) including infant stress (colour changes, tremors, startles), self-soothing capabilities, states and their organisation within the first 2 weeks after birth. The NBAS is a highly utilized measure in infant studies and has been found to have good internal consistency (Cronbach’s α = 0.94) and test-retest reliability [121]. The assessment will be separated into seven clusters for scoring purposes, with each of the NBAS items given a score (ranging from 1 to 9). Higher scores typically indicate better performance, with a lower score typically indicating poorer performance. There are some items however, in which the opposite is the case or where a midpoint score is more optimal. Each participant will be scored on each individual item using the aforementioned scales, and given a total in each of the seven clusters.

The *Still-Face Experiment (SFE)* [122] is a video-taped observational task used to assess mother-infant interaction. The SFE has been used in a wide range of development studies to assess infant affect, emotional regulation and maternal sensitivity within infant-mother dyads [123–126]. The still-face video footage will be coded on a second-by-second basis using the Infant and Caregiver Gaze by Affect/Engagement Phases (ICEP). Behaviours exhibited by mother and baby will be coded into one of nine categories. The nine ICEP categories will be collapsed into the following three categories, both for maternal and child behaviours: negative (negative engagement, hostile/intrusive, withdrawn), neutral (non-infant focused engagement), and positive (social monitor/no vocalizations or neutral vocalizations, social monitor/positive vocalizations, social positive engagement, exaggerated positive engagement). To measure dyadic matching behaviours, the proportion of time in which each mother and infant present with matched/mismatched behaviours during both the play and reunion phases will be calculated.

The *Postpartum Bonding Questionnaire (PBQ)* [127] is a 25-item self-report questionnaire that assesses maternal attitudes and feelings towards her infant. The questionnaire consists of four subscales that reflect impaired bonding, rejection and anger, anxiety about care and risk of abuse. Each of the subscales utilises a six-point scale, ranging from “always” to “never”. Responses to positive questions, such as “I enjoy playing with my baby”, will be scored from 0 (“always”) to 5 (“never”). Responses to negative questions, such as “I am afraid of my baby”, will be reverse scored from 5 (“always”) to 0 (“never”). The scores are summated, with a higher score in each of the four subscales indicating problematic bonding. The PBQ has been demonstrated to have acceptable internal consistency (Cronbach’s α = 0.76) [128].

*Infant Behaviour Questionnaire (Revised) (IBQ-R)* [129] is being used to assess infant temperament through a 36-item self-report questionnaire. Questions will be separated into six subscales to measure different temperament dimensions (smiling and laughter, distress to limitation, soothability, activity level, fear, and duration of orientation). They are answered on an eight-point scale with responses ranging from [1] never to [7] always. There is also a “does not apply” option if the event in question had not occurred at the time point of interest. Responses for each individual subscale are summed, with higher scores indicating greater levels of that temperament dimension. Each of the six subscales of the IBQ has been reported to have acceptable or good internal reliability; smiling and laughter (α = 0.85), distress to limitation (α = 0.84), soothability (α = 0.84), activity level (α = 0.73), fear (α = 0.80) and duration of orienting (α = 0.72) [129].

*Crying Patterns Questionnaire (CPQ)* [130] assesses infant crying patterns and feeding methods over 7 consecutive days and has been widely used in studies assessing crying in infants up to 9 months [130–132]. The questionnaire contains ten questions, providing information on length and frequency of crying episodes and soothing techniques for a typical day within the last week. Length and frequency of crying will be collected in hours or minutes to give a proportion of time spent crying during the day, with higher proportions indicating higher incidence of unsoothability. Frequency of soothing techniques will be scored on a four-point scale, from “never used” to “used repeatedly each day”. When compared to data collected using a diary, moderate to good convergence between maternal reports in the CPQ and diaries was found [133].

*Ages and Stages Questionnaire: Social-Emotional (ASQ-SE)* [134]*)* is a 22-item self-report questionnaire used to assess infants self-regulation, compliance, communication, autonomy, affect and interaction with people. Each question is scored on a three-point scale indicating whether the infant displays the behaviour in question [1] most of the time, [2] sometimes, or [3] never or rarely, and whether or not that particular behaviour is a concern to the parents. Each response will be given a score of 0–10, with an additional 5 points added if the parents identify a concern, and then summed. Scores can be interpreted as either above or below cut-off score (2 month = 35; 4/6 month = 45) with higher scores indicating problems with behavioural development. The ASQ has been found to have good test-retest reliability, measured as a percentage of agreement between questionnaires, found to be 94% [134]. Concurrent validity using standardized measures yielded an overall agreement of 85%, with a range of 76–91%. Internal consistency in each of the domains has been found to range between 0.49 to 0.63 when assessed at 4 months [134].

Infant sleep development will be assessed using a 24 h sleep diary adapted from Henderson [135] that will be completed by a caregiver over 2 consecutive days, once per month.

*Birth outcomes and infant anthropometry* will be assessed via participant’s medical records and will include gestational age, weight, height, head circumference, Apgar score, mode of birth, length of labour, medications during labour/birth and the postnatal period, cord clamping, type of feeding, and vitamin K injections.

#### Paternal outcome measures

**Fig. 2:**
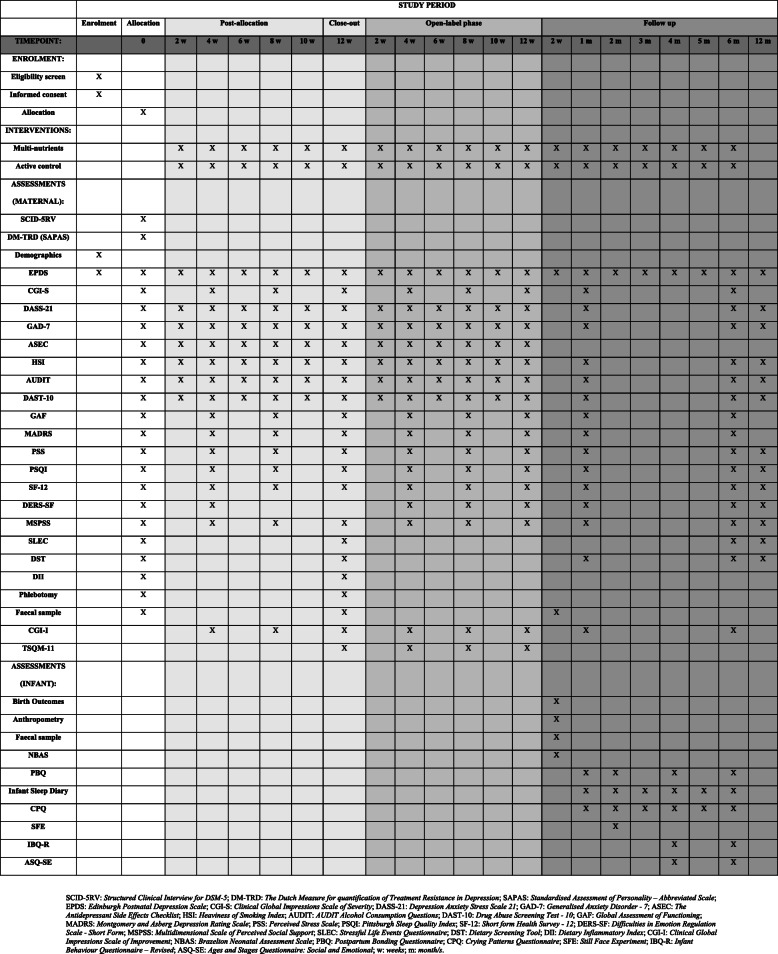
Participant flow throughout the trial

Paternal outcomes will be collected to assess potential covariates of a treatment response. Demographic characteristics will be collected and the following measures will be administered to consenting fathers at baseline, 12 weeks RCT, 1 month and 6 months postpartum: EPDS, DASS-21, SF-12 and SLEQ (Fig. 2).

### Sample size

This study aims to recruit 120 participants. In 2014, Christchurch New Zealand had 6575 live births [136]. Assuming at least 12% of pregnant women meet criteria for depression / anxiety and accounting for 1 in every 100 pregnancies being twin births, approximately 781 women per year would be eligible for the proposed study in Christchurch alone. A placebo-controlled RCT providing multinutrient supplementation to adults with ADHD [73] showed a between groups effect size of 0.64 on a measure of depression. Other RCTs found a between groups effect size of 0.39 on the EPDS when comparing a multinutrient supplement with a placebo in healthy postnatal women [137] and an effect size of 0.88 on depression outcomes from use of a B vitamin complex compared with placebo for participants with major depressive disorder [138]. Based on these findings, to detect a medium to large effect (0.6) using a two-tailed significance level of 0.05 and a power of 0.8, 45 participants per group would be needed. Assuming an attrition rate of 30% based on the mean dropout rate from the pregnant and postnatal population, 60 participants per group would provide optimal power.

### Recruitment

Participants will be recruited via social media such as Facebook; posters placed in various community settings such as midwifery, radiology and general practices, and via other media opportunities including local magazines, online news articles and the radio. Midwives and obstetricians will also be contacted and offered brochures to hand out to any potentially interested participants. Advertisements will direct prospective participants to register their interest for the study via an online screening questionnaire (http://www.bit.ly/nutrimum).

### Allocation

#### Sequence generation

A research assistant who is not involved in any other aspect of the study will generate the random allocation sequence using the following website: http://www.randomization.com. Allocation will be arranged in a 1:1 sequence using blocks of four.

#### Allocation concealment

The research assistant will seal the allocation sequence in an opaque envelope and give it to an independent pharmacist who will pre-pack the study intervention and active control accordingly. The pharmacist will seal treatment allocation cards (active or control) in the numbered opaque envelopes in accordance with the allocation sequence. The envelopes will be returned to the study site and stored in a locked drawer. This process ensures that the blind remains for all participants should the blind need to be broken for an individual participant during the course of the trial.

#### Implementation

Participants will be enrolled by the research coordinator (H.B) and will be assigned the next sequential number.

#### Blinding

Both participants and researchers including the data analyst will be blinded to the group assignment of each individual. Following analysis of the primary and secondary outcomes, the blind will be broken and participants will be informed of their treatment allocation. Breaking of the blind will be assessed on a case-by-case basis and will first be discussed with the trial physician.

### Procedure

#### Screening assessment

The online screening questionnaire will assess the eligibility of women who register their interest to participate in the study. Women who do not meet the eligibility criteria for the study will be sent an email encouraging them to speak with their lead maternity carer and consult with listed support services if needed. Women who are eligible from the screening will be contacted and asked further questions before inviting them to visit the research site to provide informed consent. Should a participant not enrol in the trial for more than 2 weeks after the initial screening or register their interest to participate in the study before 12 weeks gestation, they are required to complete a second screening to ensure they are eligible to participate in the study.

#### Informed consent

Eligible participants are sent the trial information sheet (via email or post) prior to attending the consent appointment. Researchers (HB and SC) will obtain written consent from women willing to participate in the trial following an informed discussion.

#### Baseline assessment

Following informed consent, a baseline appointment will be arranged during which participants will be asked to complete a series of online questionnaires and have their weight and blood pressure assessed. The appointment also involves an assessment of current and past mood and anxiety disorders using the Structured Clinical Interview for DSM-5, Research Version (SCID-5RV) [139]. The Dutch Measure for Quantification of Treatment Resistance in Depression (DMQTR) [117] will be utilized to predict treatment resistance. The DMQTR will be scored based on outcomes from the SCID-5RV, Global Assessment of Functioning from Axis V of the Diagnostic and Statistical Manual of Mental Disorders, Fourth Edition Text Revision [99], Standardised Assessment of Personality – Abbreviated Scale [119] and questions regarding treatment and therapy.

Participants’ partners who agree to participate in the study will provide consent separately. Participants will be given their first bottle of capsules and a capsule organizer containing vanilla sachets in each compartment in order to improve adherence and ensure consistency across treatment arms in smell. They will also be given veral and written instructions on how to take the capsules and titrate the dose over the first week. A blood form and instructions for fasting will also be given to participants who consent to provide blood samples. Participants who provide blood samples will be advised to start taking the capsules the day following blood withdrawal.

#### Trial phase and follow up

Once enrolled in the trial, participants will be required to visit the research site every 4 weeks throughout both the RCT and open label phase until their infant is born. At these visits, participants will complete assessments, collect a new bottle of capsules and return any unused capsules. The next appointment will be scheduled in order to promote participant retention. Two weeks after each visit, participants will be required to complete an online questionnaire at home that assesses mood, side effects and any adverse events. Participants will meet with the researchers for follow up assessments either at the university, at the participant’s home or if possible, over the phone (some participants may find it difficult to travel independently following a caesarean section). Assessments will occur at 1, 6 and 12 months postpartum for all participants including those who discontinue in the trial or deviate from the intervention protocol.

For each visit to the research site, participants will be given a NZD$10 petrol voucher to reimburse travel expenses. A hamper package consisting of products both bought and independently donated will also be provided to participants who complete the RCT phase of the trial in order to congratulate them on the birth of their infant and thank them for their participation. The companies who donate products are not in any other way affiliated with this research.

At the end of the open label phase, participants will decide whether or not they wish to continue taking the treatment and will accordingly be supplied with information on how to obtain the multinutrients.

### Data management

All data is collected, entered and coded by H.B and S.C and will be stored on a password protected computer system at the University of Canterbury. Electronic information will be gathered using a password protected web-based data collection system (www.canterbury.qualtrics.com) and hard copies will be stored in secure filing cabinets, behind two locked doors at the University of Canterbury.

### Data analysis

Demographic characteristics will be compared across the treatment groups using independent samples *t*-tests in order to test for potential failures of randomization.

For the primary outcomes (EPDS and CGI-I scores), the repeated measures of the outcome variables will be modelled using generalized linear mixed effects regression models using Stata 15 [140]. These models will permit the testing of differences between the multinutrient group and the active control group over the course of the trial. In the case of the EPDS, baseline scores will be used as a covariate factor. For secondary outcomes (e.g. MADRS, DASS), linear mixed effects models will also be used.

The pooled mean scores (and standard deviations) over the course of the trial on each of the primary outcomes will be used to compute estimates of effect size (Cohen’s *d*). Clinical significance between groups will be identified by a minimum of a three point difference in EPDS scores. Clinical significance within groups will be identified by a four point change on the EPDS as described by Matthey [141]. A clinical significant change will be categorised as the following: ‘Recovered’: participants who move from a score of 13 or more at baseline to 11 or less following treatment and whose score has reduced by four points; ‘Improved’: participants whose baseline score reduces by four points post treatment but is still above 13 or more; ‘Deteriorated’: participants whose baseline score increases by four points post treatment; ‘No change’: participants whose pre-post score is less than a four point change.

All data will be analysed on an intention-to-treat basis using the last observation carried forward method and as such will include all participants’ data regardless of treatment compliance. Treatment adherence will be measured by the total number of capsules consumed throughout the trial phases. Only participants who consume 70% or more of the total number of capsules will be considered adherent to the treatment. A per-protocol analysis, which excludes participants with protocol violations (e.g. drop out, non-adherence to treatment consumption) will be performed on the primary outcomes (EPDS and CGI-I) in order to compare differences in treatments responses between participants who were adherent with the treatment and those who discontinued participation or were non-adherent. Outcomes from the open label phase will be compared to the group outcomes from the week 12 observation of the RCT phase using generalised linear mixed effects regression models taking into account participants self-reported use of micronutrients.

To assess the effect of the multinutrient intervention has on infant development, we will compare those infants exposed to the multinutrients versus those infants not exposed to the multinutrients on emotional, cognitive and physical development. Length of exposure to the multinutrients (due to the RCT phase and varying starting points during gestation) and score on the EPDS will also be considered as continuous variables in considering infant outcomes. Hierarchical regression analysis will be run using Stata 15 in order to identify if perinatal/postpartum depression and multinutrient exposure are predictors of improved self-regulation of the infant across the various time points. Descriptive statistics for each participant will be used to compare demographic characteristics between the treatment groups to allow any differences to be controlled.

### Data monitoring

Trial progress and adverse events will be reported and assessed by the Southern Human and Disabilities Ethics Committee every 12 months. The trial will be terminated if serious adverse effects known to be caused by the nutrients occur and/or if there is no evidence of any improvement in symptoms in the first 40 participants who complete the RCT phase of the trial. An interim analysis will not be conducted.

### Safety monitoring and reporting

A protocol is in place to respond to situations in which a participant reports adverse symptoms of either a physical or psychological nature throughout the duration of the trial. Adverse events and side effects will be monitored within the first week of the trial via telephone and fortnightly thereafter via a questionnaire asking participants to identify any new medical problems, infections, hospitalizations (excluding birth) and medication and supplement use. Participants will be advised to contact the study investigators if they are concerned about any increases in physical or psychological symptoms.

Should a participant’s psychological symptoms worsen, they will be instructed to contact the study investigators who in discussion with the participant, may refer them to their General Practitioner (GP), psychiatric emergency services or may decide to offer the open label option earlier if there is a worsening in symptoms during the RCT phase. If a participant’s psychological state deteriorates to a clinically significant degree during the trial, the investigators will discuss with the participant the possibility of withdrawing from the study, or may decide that the participant should be withdrawn. If withdrawn, participants will be referred to health care services as appropriate. Participants will also be informed that they can withdraw from the trial for any reason at any time without penalty. Should a participant withdraw or be withdrawn from the trial or be lost to follow up, relevant details will be documented. In the event of a physical or psychiatric emergency, participants will be advised to contact emergency services or visit the emergency department immediately. Participants incurring a physical injury as a result of participating in the trial will be eligible for compensation from Accident Compensation Corporation (ACC) under the Accident Compensation Act 2001 [142].

Serious adverse events will be categorized according to the New Zealand Ministry of Health guidelines. According to the New Zealand Ministry of Health, a serious adverse event is considered to occur at any dose that results in death, or is life threatening, or requires inpatient hospitalization or prolongs hospitalization, or results in persistent or significant disability or incapacity, or is a congenital anomaly or birth defect [143]. Any serious adverse events will be recorded and reported annually to the Southern Health and Disability Ethics Committee, New Zealand.

### Study integrity

This study has received ethical approval by the Southern Human and Disabilities Ethics Committee (Reference: 16/STH/187) on 3 February 2017 and the Standing Committee on Therapeutic Trials (Reference: 16/SCOTT/131) on 7 February 2017. The study has also received approval from the New Zealand College of Midwives and the Human Ethics Committee and Ngā Tahu Consultation and Engagement Group at the University of Canterbury. The Universal Trial Number (UTN) for this study is U1111–1189-4070. Any protocol amendments will be raised with the ethics committee for approval and approved changes will be updated on the trial registry and in the trial protocol. Protocol version: 06; 23 January 2019.

This trial was prospectively registered in the Australian and New Zealand Clinical Trials Registry (ANZCTR) on 8 March 2017 (Trail ID: ACTRN12617000354381) (Table 2).

**Table 2:** Trial registration data

Data category	Trial information
Primary registry and trial identification number	Australian and New Zealand Clinical Trials Registry; ACTRN12617000354381
Date of registration in primary registry	8 March 2017
Secondary identifying numbers	Universal Trial Number (UTN): U1111–1189-4070
Source(s) of monetary or material support	Hardy Nutritionals, Department of Psychology Research Funds, University of Canterbury; University of Canterbury Foundation; Foundation for Excellence in Mental Health Care; The Nurture Foundation for Reproductive Research; St George’s Hospital, NZ, Canterbury Medical Research Foundation,
Primary sponsor	Prof Julia Rucklidge
Secondary sponsor(s)	Prof Roger Mulder, Dr. Jacqueline Henderson, Prof Martin Kennedy, Dr. Kyle Nash, Dr. Lesley Dixon, A/P Joseph Boden, Hayley Bradley
Contact for public enquiries	Hayley Bradley, hayley.bradley@pg.canterbury.ac.nz
Contact for scientific enquiries	Prof Julia Rucklidge, julia.rucklidge@canterbury.ac.nz
Public title	A multinutrient intervention for pregnant women experiencing symptoms of depression and anxiety.
Scientific title	An investigation examining the efficacy and safety of a multinutrient intervention on symptoms of antenatal depression and anxiety in pregnant women who are symptomatic: A double blind, randomized, controlled trial.
Countries of recruitment	New Zealand
Health condition(s) or problem(s) studied	Depression, anxiety
Interventions	Intervention: Daily Essential Nutrients (12 capsules per day)
	Active control: Iodine and Riboflavin (12 capsules per day)
Key inclusion and exclusion criteria	Ages eligible for study: ≥16 years; Sexes available for study: femaleAccepts healthy volunteers: no
	Inclusion criteria: women aged 16 years and over; 12–24 weeks gestation; low risk singleton pregnancy; free from psychiatric medication for four weeks; score of 13 or more on the Edinburgh Postnatal Depression Scale (EPDS); deemed reliable and compliant with the protocol
	Exclusion criteria: regular vomiting; high risk pregnancy; significant pregnancy complications; known foetal abnormalities; serious current or historical medical condition; known allergy to the ingredients of the intervention; known metabolic condition such as Wilson’s disease, hemochromatosis.; untreated or unstable thyroid disease; known neurological disorder; desire to continue taking prenatal supplements that either exceed the upper limit or are not required for medical purposes (decisions discussed and made on a case-by-case basis)
Study type	Interventional
	Allocation: randomised, parallel assignment; Masking: blinding of people taking and administering the treatment, assessing the outcomes and analysing the results / data
	Primary purpose: treatment
Date of first enrolment	12 April 2017
Target sample size	120
Recruitment status	Recruiting
Primary outcome(s)	Depression and anxiety; Edinburgh Postnatal Depression Scale (time points: screening, baseline, every two weeks until birth and at 1 month and 6 months postpartum)
	Symptom improvement; Clinical Global Impressions Scale – Improvement (time points: baseline, every four weeks until birth and at 1 month and 6 months postpartum)
Key secondary outcomes	Depression; The Montgomery and Asberg Depression Rating Scale (time points: baseline, every four weeks until birth and at 1 month and 6 months postpartum)
	Anxiety; Generalized Anxiety Disorder – 7 (time points: baseline, every two weeks until birth and at 1 month and 6 months postpartum)
	Depression, Anxiety, Stress; Depression, Anxiety, Stress Scale – 21 (time points: baseline, every two weeks until birth and at 1 month and 6 months postpartum)
	Sleep quality; The Pittsburgh Sleep Quality Index (time points: baseline, every four weeks until birth and at 1 month and 6 months postpartum)
	Quality of life; Short Form Health Survey – 12 (time points: baseline, every four weeks until birth and at 1 month and 6 months postpartum)
	Emotion dysregulation; Difficulties in Emotion Regulation Scale - Short Form (time points: baseline, every four weeks until birth and at 1 month and 6 months postpartum)
	Side effects; Antidepressant Side-Effect Checklist, (time points: baseline, every two weeks until birth and at 1 month and 6 months postpartum)
	Plasma nutrient levels (vitamin C, vitamin B12, vitamin D, copper, zinc, iron and homocysteine) (time points: baseline and 12 weeks)
	Inflammatory biomarkers (interleukin – 6, tumor necrosis factor - alpha, interleukin-4 and interleukin-10) (time points: baseline and 12 weeks)
	Anxiety related endophenotypes and neurophysiological markers; Electroencephalography (time points: baseline and 12 weeks)
	Microbiome; faecal samples (time points: baseline and 12 weeks)

All participants will be required to provide informed consent before entry into the study and any personal details will be stored in locked filing cabinets and password protected databases. Study records and the final trial dataset will identify participants by their ID number only. Only the investigators affiliated with this trial will have access to the final dataset. The trial results will be released to participants and health professionals and other referring agents including a mothers and babies psychiatric service and obstetric and midwifery practices. Findings will also be published in peer-reviewed journals and disseminated via social media and other media outlets.

## Discussion

Pregnant women suffering from depression and anxiety have limited treatment options available for their symptoms. This RCT with an open label phase and naturalistic follow up has been designed to assess the efficacy and safety of a multinutrient formula on symptoms of mood and anxiety during antenatal period and the impact in-utero exposure to multinutrients may have on infant development. Should the nutrients be shown to be safe and beneficial, then we begin to build an evidence base of an alternative treatment option for women suffering from antenatal depression and anxiety. The findings may also improve the short and long-term outcomes not only for the mother and the infant but also for their families and community.

## Data Availability

Not applicable.

## Abbreviations

RCT: Randomised controlled trial
OL: Open label
DEN: Daily Essential Nutrients
CES-D: Centre for Epidemiologic Studies – Depression Scale
ADHD: Attention deficit hyperactivity disorder
ASD: Autism spectrum disorder
EPDS: Edinburgh Postnatal Depression Scale
CGI-I: Clinical Global Impressions – Improvement Scale
CGI-S: Clinical Global Impressions Scale – Severity Scale
SCID-5RV: Structured Clinical Interview for DSM-5 (research version)
GAF: Global Assessment of Functioning
MADRS: Montgomery and Asberg Depression Rating Scale
DM-TRD: The Dutch Measure for Quantification of Treatment Resistance in Depression
SAPAS: Standardised Assessment of Personality - Abbreviated Scale
DASS-21: Depression, Anxiety, Stress Scale - 21
GAD-7: Generalized Anxiety Disorder - 7
PSS: Perceived Stress Scale
PSQI: Pittsburgh Sleep Quality Index
SF-12: Short form Health Survey - 12
DERS-SF: Difficulties in Emotion Regulation Scale - Short Form
ASEC: Antidepressant Side-Effect Checklist;
MSPSS: Multidimensional Scale of Perceived Social Support
SLEQ: Stressful Life Events Questionnaire
HSI: Heaviness of Smoking Index
AUDIT: AUDIT Alcohol Consumption Questions
DAST-10: Drug Abuse Screening Test - 10
DST: Dietary Screening Tool
DII: Dietary Inflammatory Index
TSQM-11: Treatment Satisfaction Questionnaire for Medication - 11
NBAS: Brazelton Neonatal Assessment Scale
PBQ: Postpartum Bonding Questionnaire
CPQ: Crying Patterns Questionnaire
SFE: Still Face Experiment
IBQ-R: Infant Behaviour Questionnaire – Revised
ASQ-SE: Ages and Stages Questionnaire: Social and Emotional
ACC: Accident Compensation Corporation
UTN: Universal Trial Number
